# Patterns of diversification amongst tropical regions compared: a case study in Sapotaceae

**DOI:** 10.3389/fgene.2014.00362

**Published:** 2014-12-03

**Authors:** Kate E. Armstrong, Graham N. Stone, James A. Nicholls, Eugenio Valderrama, Arne A. Anderberg, Jenny Smedmark, Laurent Gautier, Yamama Naciri, Richard Milne, James E. Richardson

**Affiliations:** ^1^The New York Botanical GardenBronx, NY, USA; ^2^Institute of Evolutionary Biology, University of EdinburghEdinburgh, Scotland; ^3^Royal Botanic Garden EdinburghEdinburgh, Scotland; ^4^Naturhistoriska RiksmuseetStockholm, Sweden; ^5^University Museum of BergenBergen, Norway; ^6^Conservatoire et Jardin botaniquesGenève, Switzerland; ^7^Institute of Molecular Plant Sciences, University of EdinburghEdinburgh, Scotland; ^8^Laboratorio de Botánica y Sistemática, Universidad de los AndesBogotá DC, Colombia

**Keywords:** Sapotaceae, *Manilkara*, pantropical, biogeography, diversification rates

## Abstract

Species diversity is unequally distributed across the globe, with the greatest concentration occurring in the tropics. Even within the tropics, there are significant differences in the numbers of taxa found in each continental region. *Manilkara* is a pantropical genus of trees in the Sapotaceae comprising *c*. 78 species. Its distribution allows for biogeographic investigation and testing of whether rates of diversification differ amongst tropical regions. The age and geographical origin of *Manilkara* are inferred to determine whether Gondwanan break-up, boreotropical migration or long distance dispersal have shaped its current disjunct distribution. Diversification rates through time are also analyzed to determine whether the timing and tempo of speciation on each continent coincides with geoclimatic events. Bayesian analyses of nuclear (ITS) and plastid (*rpl32-trnL, rps16-trnK*, and *trnS-trnFM*) sequences were used to reconstruct a species level phylogeny of *Manilkara* and related genera in the tribe Mimusopeae. Analyses of the nuclear data using a fossil-calibrated relaxed molecular clock indicate that *Manilkara* evolved 32–29 million years ago (Mya) in Africa. Lineages within the genus dispersed to the Neotropics 26–18 Mya and to Asia 28–15 Mya. Higher speciation rates are found in the Neotropical *Manilkara* clade than in either African or Asian clades. Dating of regional diversification correlates with known palaeoclimatic events. In South America, the divergence between Atlantic coastal forest and Amazonian clades coincides with the formation of drier Cerrado and Caatinga habitats between them. In Africa diversification coincides with Tertiary cycles of aridification and uplift of the east African plateaux. In Southeast Asia dispersal may have been limited by the relatively recent emergence of land in New Guinea and islands further east *c*. 10 Mya.

## Introduction

Biodiversity is unevenly distributed across the globe and is most intensely concentrated in the tropics, particularly in wet tropical forests, which are the most species-rich biomes on the planet. Even within the tropics, there are significant differences in the floristic composition and the numbers of taxa found in each of the continental regions. It is estimated that there are *c*. 27,000 species of flowering plants in tropical Africa (Lebrun, 2001; Lebrun and Stork, 2003), compared with *c*. 90,000 for South America (Thomas, 1999) and *c*. 50,000 for Southeast Asia (Whitmore, 1998). This uneven species diversity raises the fundamental question of how variation in the pattern and tempo of speciation and extinction among continents might have driven observed patterns. Differences in diversity have been attributed to higher extinction rates in Africa (Richards, 1973) and faster diversification in the Neotropics (Gentry, 1982). Dated molecular phylogenies suggest speciation in response to recent climatic changes (such as aridification, e.g., Couvreur et al., 2008; Simon et al., 2009) or geological phenomena (such as mountain uplift in the Neotropics, e.g., Richardson et al., 2001; Hughes and Eastwood, 2006).

Intercontinental disjunctions in distribution between tropical regions of Africa, Asia and South America have been attributed to Gondwanan break-up (Raven and Axelrod, 1974), and/or the degradation of the boreotropical flora (e.g., Malpighiacaeae, Davis et al., 2002b; Meliaceae, Muellner et al., 2006; Moraceae, Zerega et al., 2005). However, current studies have shown that many tropical groups are of more recent origin (e.g., *Begonia*, Thomas et al., 2012), and that long distance dispersal has been an important factor in determining the composition of modern tropical floras (Pennington et al., 2006; Christenhusz and Chase, 2013). While long-distance dispersal could have occurred at any time, it was generally believed to be the only viable explanation for tropical intercontinental disjunctions younger than *c*. 33 Mya (although see Zhou et al., 2012).

Pantropically distributed taxa are excellent models for studying the evolution of tropical forests and regional variation in diversification rates between continents. *Manilkara* is a genus of trees in the Sapotaceae comprising *c*. 78 species distributed throughout the tropics (30 in South and Central America, 35 in Africa and 13 in Southeast Asia). This even spread and relatively low number of species across major tropical regions makes *Manilkara* an excellent candidate for comparison of regional diversification patterns and testing of hypotheses for the genesis of pantropical distributions. Here a near species-level dated phylogeny of *Manilkara* is presented. If the distribution of the genus can be explained by Gondwanan break up, the timing of phylogenetic splits would be expected to reflect that break up 165–70 Mya (McLoughlin, 2001). Similarly if splits resulted from the degradation of the boreotropical flora, they would be expected to occur as temperatures cooled following the Early Eocene Climatic Optimum/Paleocene–Eocene Thermal Maximum (EECO/PETM), 50–55 Mya (Zachos et al., 2001). Additionally, a boreotropical origin should leave a phylogeographic signature in the form of southern lineages being nested within more northern ones. Therefore, lineages in South America or to the east of Wallace's Line would be nested within Laurasian lineages, resulting in the pattern one would expect from a retreat of the boreotropical flora from the Northern Hemisphere. The onset of glaciation from 33 Mya induced further global cooling (Zachos et al., 2001) and the disintegration of the boreotropical flora. Therefore, ages of splits younger than *c*. 33 Mya would most likely be explained by long distance dispersal. The prediction advanced by Gentry (1982) that diversification rates in the Neotropics have been higher than in other tropical regions is also tested.

## Materials and methods

### DNA extraction, PCR, sequencing, and alignment

Evolutionary relationships were reconstructed using nuclear (ITS) and plastid (*rpl32-trnL, rps16-trnK*, and *trnS-trnFM*) sequences. Divergence times were calculated using an ITS dataset with 171 accessions of Sapotaceae. In total 53 of the global total of 79 *Manilkara* species (67%) were included in the analysis. The dataset includes representatives of the tribe Mimusopeae as well as multiple representatives of the tribes Isonandreae and Sideroxyleae, which also belong to the subfamily Sapotoideae, in order to accommodate calibration of fossils related to those groups. The tree was rooted using *Sarcosperma*, shown in previous studies to be sister to the rest of the family (Anderberg and Swenson, 2003). The plastid dataset comprised 95 accessions of subtribe Manilkarinae, as well as outgroups in subtribe Mimusopinae, plus *Northia, Inhambanella, Eberhardtia*, and *Sarcosperma*, which provided the root for the tree. See Supplementary Table 1 for the list of taxa with voucher specimen information and GenBank accession numbers.

Total DNA was extracted from herbarium specimens and silica gel-dried leaf samples using the Qiagen Plant DNeasy Mini Kit following the manufacturer's instructions. Amplifications of the ITS region were performed using the ITS5p/ITS8p/ITS2g/ITS3p (Möller and Cronk, 1997) and ITS1/ITS4 (White et al., 1990) primer pairs. Polymerase chain reaction (PCR) was carried out in 25-μL volume reactions containing 1 μL of genomic DNA, 5.75 μL sterile distilled water, 2.5 μL 2 mM dNTPs, 2.5 μL 10x NH_4_ reaction buffer, 1.25 μL 25 mM MgCl_2_, 0.75 μL of each 10 μM primer, 10 μL 5 M betaine, 0.25 μL BSA and 0.25 μL of 5 u/μL Biotaq DNA polymerase buffer. The thermal cycling profile consisted of 5 min denaturation at 95°C, followed by 35 cycles of 30 s at 95°C for denaturation, 50°C for 30 s for annealing and 72°C for 1 min and 30 s for extension with a final extension period of 8 min at 72°C on a Tetrad2 BioRad DNA Engine. Extraction from herbarium specimens often yielded low amounts of degraded DNA and required nested PCR to amplify quantities sufficient for sequencing. In nested PCR the ITS5/ITS8 primer pair was used in the first reaction. 1 μl of this PCR product was then used in a second PCR with the ITS1/ITS4 primer pair and the same thermocycling profile. Further internal primers, ITS2g and ITS3p, were used in place of ITS1 and ITS4 when amplification using the latter primers was unsuccessful. Plastid markers were amplified using *rpl32-trnL* (Shaw et al., 2007), *rps16-trnK* (Shaw et al., 2007), and *trnS-trnFM* (Demesure et al., 1995) primer pairs as well as *Manilkara*-specific internal primers designed for this study (Supplementary Table 2). PCR was carried out in 25 μL volume reactions containing 1 μL of genomic DNA, 15.25 μL sterile distilled water, 2.5 μL 2 mM dNTPs, 2.5 μL 10x NH_4_ reaction buffer, 1.25 μL 25 mM MgCl_2_, 0.75 μL of each 10 μM primer, 0.8 μL BSA and 0.2 μL of 5 u/μL Biotaq DNA polymerase buffer. All plastid regions were amplified using the *rpl16* program of Shaw et al. (2005). Nested PCR was also performed on selected accessions using self-designed internal primers (Supplementary Table 2). PCR products were purified using Exo-SAP (GE Healthcare) according to the manufacturer's instructions.

Sequencing PCRs were carried out using the BigDye Terminator v. 3.1 Cycle Sequencing Kit (Applied Biosystems) and were purified and sequenced on an ABI 3730 sequencer at the University of Edinburgh's GenePool facility. Forward and reverse sequences were assembled into contiguous sequences (contigs) and edited using the alignment software Sequencher ver. 4.7. Edited contigs were assembled and aligned by eye in MacClade ver. 4.08 (Maddison and Maddison, 2008) and later in BioEdit ver. 7.0.5 (Hall, 2005).

Potentially informative indels in the plastid dataset were coded according to the simple indel coding method of Simmons and Ochoterena (2000). Ambiguous alignment regions 113–118 and 380–459 in *rps16-trnK* were excluded. Indel events in ITS were so frequent that their coding as additional characters was deemed to be too ambiguous. Gaps were treated as missing data and all characters were equally weighted.

The ITS dataset was partitioned into three segments: ITS1 (372 bp), 5.8 s (167 bp), and ITS2 (339 bp). Plastid regions and their indels were retained as separate partitions: *rpl32-trnL* (1130 bp + 26 indels), *rps16-trnK* (1134 bp + 21 indels), and *trnS-trnFM* (999 bp + 13 indels).

### Phylogenetic analysis

Bayesian analyses were carried out using MrBayes 3.1 (Huelsenbeck and Ronquist, 2001). Two independent runs of four Metropolis Coupled Monte Carlo Markov Chains (MCMCMC) each (three heated and one cold) were run with a temperature setting of 0.10 for 8,000,000 generations, which was found to provide sufficient mixing between chains and convergence between runs. Trees were sampled every 8000 generations and a 10% burn-in was removed from the sampled set of trees leaving a final sample of 900 trees, which were used to produce a majority rule consensus tree. Convergence of models was determined to have occurred when the standard deviation of split frequencies for two runs reached 0.01 (Ronquist et al., 2005). Appropriate burn-in and model convergence were checked by visual confirmation of parameter convergence of traces in Tracer v.1.5 (Rambaut and Drummond, 2009). Clade support values are posterior probabilities (pp); pp values of 100–95% are taken to indicate strong support, values of 94–90% moderate support, and values between 89 and 55% weak support for nodes, respectively. The output tree files were visualized in FigTree v.1.3.1. The majority rule consensus tree was used to determine the monophyly of key clades used to define calibration points in the dating analysis.

Plastid data were not included in the subsequent BEAST analysis because they were not informative enough to discern between alternative hypotheses and because fewer taxa were sampled. Additionally, hard incongruence was demonstrated between the topologies reconstructed in MrBayes from the nuclear and plastid datasets (see Supplementary Material Section on chloroplast capture, and Supplementary Figure 1). Therefore, the two datasets were not combined and only nuclear data was used for divergence time analysis.

### Fossil calibration

**Figure 1:**
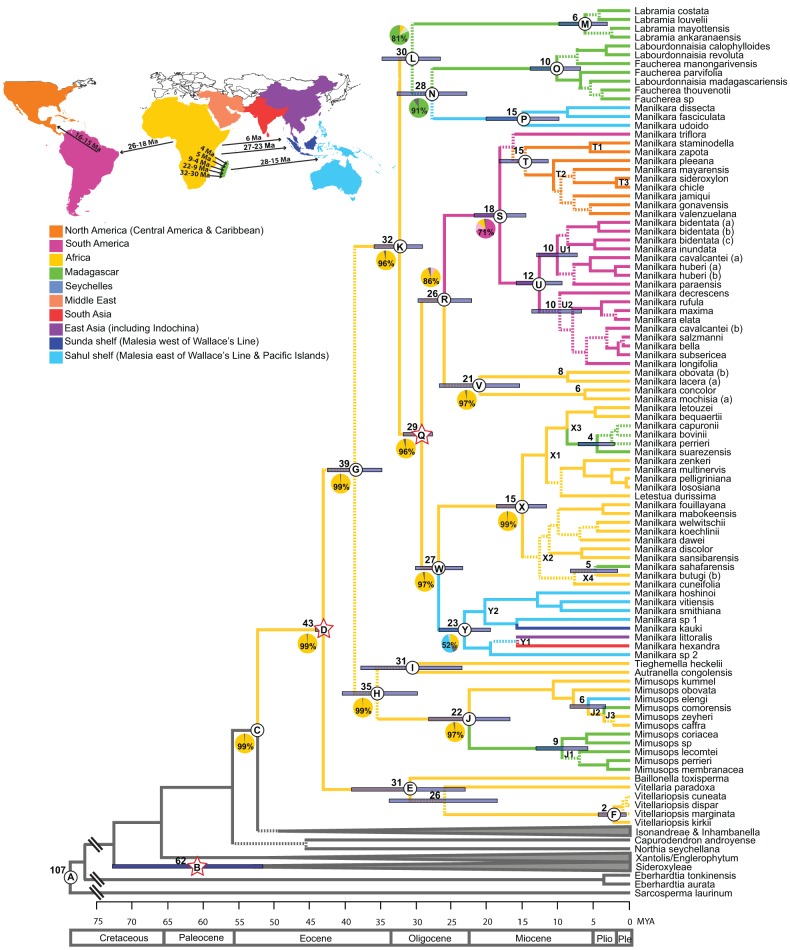
**Maximum clade credibility chronogram of the ITS dataset**. Dashed lines indicate branches which lead to nodes with a posterior probability of <0.95. Mean ages are given for profiled nodes. Node bars indicate 95% HPD age ranges. Lettered nodes are discussed in the text. Stars indicate the placement of fossils. Lineages are colored according to their distribution: Yellow, Africa; Green, Madagascar; Blue, Asia; Pink, South America; Orange, Central America and the Caribbean. Geological epochs are indicated in a scale at the bottom of the chronogram. Outgroups have been reduced to gray bars at the base of the chronogram. Ten regions were coded in the ancestral area reconstruction as illustrated in the map and legend. Pie charts represent the percentage likelihood of the ancestral state at the selected node. Map inset depicts the timing and direction of long-distance dispersal events reflected in the chronogram.

Sideroxyleae pollen from the Ypresian (47.8–56 Mya) of England (Gruas-Cavagnetto, 1976) was used to constrain the minimum age of the Sideroxyleae stem node (node B in Figure 1). A log normal prior was used to constrain the age of this node (offset: 52.2 Ma, mean: 0.001). A mean of 0.001 was chosen so that 95% of the probability is contained in an interval between the midpoint and the upper boundary of the Ypresian (52.2–55.6 Mya). A Mid-Eocene (37.2–48.6 Mya) *Tetracolporpollenites* pollen grain from the Isle of Wight was used to constrain the minimum age of the node for the tribe Mimusopeae. This pollen grain was described by Harley (1991) and determined to closely resemble *Tieghemella heckelii* (a monotypic genus in the Mimusopeae). Harley suggested (pers. comm. 2010) that it would be appropriate to err on the side of caution with the identification and use the fossil to constrain the age of the tribe Mimusopeae rather than the genus itself. This fossil was, therefore, used to constrain the age of the crown node of Mimusopeae (node D in Figure 1: offset: 42.9 Mya, mean: 0.095). A mean of 0.095 was chosen so that 95% of the probability was contained in an interval between the midpoint (42.9) and the upper boundary of the mid Eocene (42.9–48.6 Mya). The final calibration point is based on a series of Oligocene (23–33.9 Mya) fossil leaves from Ethiopia (Jacobs et al., 2005). Pan described these specimens as *Sapoteae* sp. and suggested possible placement in either *Manilkara* or *Tieghemella* (pers. comm. 2010) based on the occurrence of stoma surrounded by fimbricate periclinal rings, a character present in these genera, but absent from the related genera *Autranella* and *Mimusops*. Although they are both members of the Tribe Mimusopeae, *Manilkara* and *Tieghemella* are not sister taxa, and placing the fossil at the node of the most recent common ancestor (the entire Tribe Mimusopeae) seemed illogical for such a young date, when a 45 Mya fossil pollen grain of cf. *Tieghemella* was a better fit for the same node. Instead, the fossil was alternatively placed at the *Manilkara* crown node (node Q in Figure 1) and on the node of the split between *Tieghemella* and *Autranella* (node I in Figure 1), in order to determine whether placement on either genus made a significant difference to age estimates using a prior age estimate with an offset of 28 Mya, mean: 0.1. A mean of 0.1 was chosen so that 95% of the probability was contained in an interval between the midpoint and the upper boundary of the Oligocene at (28-33.9 Mya).

### Dating analysis

The software package BEAST v.1.7.5 (Drummond and Rambaut, 2007) was used to analyze divergence times in the ITS dataset. An xml input file was created in BEAUti v.1.7.5. Substitution models were unlinked across partitions, but clock models and tree topologies were kept on the linked default setting. Four taxon sets per analysis were generated in order to define nodes for placement of fossil calibration points. They were based on known monophyletic clades from previous analyses and were constrained to be monophyletic.

The GTR + I + G model was applied to each partition. The mean substitution rate was not fixed and base frequencies were estimated. Following support for a molecular clock in these data using MrBayes, an uncorrelated log-normal model was selected to allow for relaxed clock rates and rate heterogeneity between lineages. A speciation: birth-death process tree prior was used with a randomly generated starting tree. The most recent common ancestor (MRCA) node age priors were set to define calibration points using taxon sets. All other priors were left at default settings that were either uniform or gamma-distributed. Posterior distributions for each parameter were estimated using MCMCMC run for 40,000,000 generations, with parameters logged every 5000 generations, giving 8000 samples per run. The BEAUti xml file was executed in BEAST v.1.7.5. Two separate analyses were run and the output log files were reviewed in Tracer v.1.5 (Rambaut and Drummond, 2009) to check for convergence between runs and adequate effective sampling sizes (ESS) of >200 (Drummond et al., 2007). The tree files from the two runs were combined in LogCombiner v.1.7.5 (Drummond and Rambaut, 2007) with a conservative burn-in of 4000 generations. The combined tree files were input into TreeAnnotator v.1.5.3 (Drummond and Rambaut, 2007). The Maximum Clade Credibility (MCC) tree was selected with mean node heights; this option summarizes the tree node height statistics from the posterior sample with the maximum sum of posterior probabilities. The output file was visualized in FigTree v.1.3.1.

### Ancestral area reconstruction in RASP

Ancestral area states were reconstructed in RASP (Reconstruct Ancestral State in Phylogenies; http://mnh.scu.edu.cn/soft/blog/RASP) software that implements Bayesian Binary MCMC (BBM) time-events curve analysis (Yu et al., 2011) and allows multiple states to be assigned to terminals. BBM suggests possible ancestral ranges at each node and also calculates probabilities of each ancestral range at nodes. The analysis was performed using the MCC tree generated in BEAST as an input file, with 5,000,000 cycles, 10 chains, sampling every 100 cycles, with a temperature setting of 0.1 and with the maximum number of areas set to four for all nodes. The root node was defined *a priori* as Asian; because the Asian taxa *Sarcosperma* and *Eberhardtia* form a grade within which the rest of the family is nested, this is the most likely state for the crown node of the family.

Areas are coded according to continent, based predominantly on tectonic plate margins and then on floristic regions (Figure 1). In Southeast Asia, the Sahul and Sunda Shelves (which mark the boundary between continental Asia and Australia-New Guinea) were coded as separate states within the Malesia floristic region, which stretches from the Isthmus of Kra on the Malay Peninsula to Fiji (Takhtajan, 1986; Van Welzen et al., 2005). East Asia is defined as being east of the Himalayas and south as far as the Malay Peninsula, with a predominantly Indo-Chinese flora. South Asia is delineated by the margin of the Indian subcontinent. The countries of Iran, Turkey and the Arabian Peninsula support a drier Irano-Turanian flora (Takhtajan, 1986) and were, therefore, designated as being part of the Middle-Eastern region. The remaining regions (the Seychelles, Madagascar, Africa and North and South America) are all on separate continental tectonic plates and are floristically unique from one another (see Supplementary Table 1 for species-specific area codes).

### Diversification rate methods

A separate ITS lineage through time (LTT) plot dataset (hereafter referred to as ITS LTT) was used to compare diversification rates within *Manilkara*. Because the genus was found to be paraphyletic, with the Southeast Asian *M. fasciculata* clade (P in Figure 1) being more closely related to *Labourdonnaisia* and *Faucherea*, this small clade was excluded, leaving only the monophyletic lineage of *Manilkara s.s*. (clade Q in Figure 1) for analysis. Additionally, only one individual per species was included. The simple diversification rate estimators of Kendall (1949) and Moran (1951) were calculated for the African, Neotropical and Asian clades, where the speciation rate lnSR = [ln(N)−ln(N_0_)]/T (N = standing diversity, N_0_ = initial diversity, here taken as = 1, and T = inferred clade age). This is a pure-birth model of diversification with a constant rate and no extinction (Magallón and Sanderson, 2001). Another model that does not assume constant rates of speciation and extinction through time within lineages was applied using BAMM (Bayesian Analysis of Macroevolutionary Mixtures; Rabosky, 2014). BAMM uses a reversible-jump Markov Chain Monte Carlo to explore shifts between macroevolutionary regimes, assuming they occur across the branches of a phylogenetic tree under a compound Poisson process. Each regime consists of a time-varying speciation rate (modeled with an exponential change function) and a constant rate of extinction. The BAMM analysis used the BEAST MCC tree, but because not all species were sampled, it was necessary to specify to which lineage each of the missing taxa belonged (i.e., to which species it was most closely related based on morphological similarity). The results of the analysis with adjustments to account for missing taxa were not different from those assuming complete taxon sampling. Two MCMC simulations were run with 5,000,000 generations, sampling every 1000, and discarding the first 10% as burn-in. Appropriate priors for the ITS LTT phylogeny, convergence of the runs and effective sampling size were each estimated using the BAMMtools (Rabosky, 2014) package in R (R development team).

LTT plots were generated using phytools (Revell, 2012) in R for 1000 trees sampled through the post-burn-in (20%) posterior distribution generated by BEAST (see above for details). The median and 95% highest posterior density (HPD) were estimated for the ages of each number of lineages in each plot. To compare the observed LTT plots with the predictions of a model with constant diversification rates, 1000 trees were simulated using the mean speciation and extinction rates estimated by BAMM in TreeSim (Stadler, 2011). Simulations used the age of the most recent common ancestor of each of the 1000 observed trees and the current number of species per plot. LTT plots were drawn for the trees including all species of *Manilkara s.s*. and to examine region-specific patterns for pruned lineages that included only those species from each of Africa, the Neotropics and Asia.

## Results

### Node ages

Mean ages with 95% HPD confidence intervals for key nodes are reported in Table 1. The MCC tree from the BEAST analysis (Figure 1) resolves the mean crown age of the tribe Mimusopeae as 43 Mya (HPD 44–42 Mya; node D), in the Mid Eocene. The mean age of subtribe Manilkarinae is estimated to be 32 Mya (HPD 36–29 Mya; node K) and the genus *Manilkara* is resolved as 29 Mya (HPD 32–28 Mya; node Q), both having originated during the Oligocene. Results also reveal that cladogenesis and inter-continental dispersal (see below and Figures 1, **3**) within *Manilkara* occurred from the Oligocene through the Miocene—and most intensively from the mid-late Miocene.

**Table 1:** **Summary of clade support values, node ages and ancestral areas from Figure 1**.

**Node**	**Posterior probability**	**Clade**	**Mean age and 95% HPD in Mya**	**Ancestral Area (likelihood %)**	**Epoch**
A	1	Sapotaceae	107 (126-88)	East Asia 99	Cretaceous
B	1	Sideroxyleae	62 (73-52)	Africa 58	Cretaceous-Paleocene
C	0.99	Isonandreae/*Inhambanella*/Mimusopeae	52 (58-48)	Africa 99	Paleocene-Eocene
D	1	Mimusopeae	43 (44-42)	Africa 99	Eocene
E	0.99	*Baillonella/Vitellaria/Vitellariopsis*	31 (39-23)	Africa 99	Eocene-Oligocene
F	0.99	*Vitellariopsis*	2 (4-0.5)	Africa 99	Pliocene
G	0.85	Mimusopeae subclade 1	39 (43-35)	Africa 99	Eocene
H	0.67	*Mimusops/ Tieghemella/Autranella*	35 (40-30)	Africa 99	Eocene-Oligocene
I	0.68	*Tieghemella/Autranella*	31 (38-23)	Africa 99	Eocene-Oligocene
J	0.99	*Mimusops*	22 (28-17)	Africa 97	Miocene
K	0.99	Manilkarinae	32 (36-29)	Africa 96	Eocene-Oligocene
L	0.44	*Labr./Fauch./Labourd./*sm. Asian *Manilkara*	30 (35-26)	Madagascar 81	Eocene-Oligocene
M	0.99	*Labramia*	6 (10-3)	Madagascar 99	Miocene-Pliocene
N	0.92	*Faucherea/Labourdonnaisia/Manilkara*	28 (33-23)	Madagascar 91	Oligocene
O	0.99	*Faucherea/Labourdonnaisia*	10 (14-7)	Madagascar 99	Miocene-Pliocene
P	0.99	Small Asian *Manilkara*	15 (20-10)	Sahul Shelf 90	Miocene
Q	1	*Manilkara s.s*.	29 (32-28)	Africa 96	Oligocene
R	0.98	*Manilkara s.s*. subclade 1	26 (30-22)	Africa 86	Oligocene-Miocene
S	0.99	Neotropical *Manilkara*	18 (22-14)	South America 71	Miocene
T	0.90	Central American and Caribbean *Manilkara*	15 (20-13)	North America 95	Miocene
U	0.99	South American *Manilkara s.s*.	12 (16-9)	South America 93	Miocene
V	0.77	Small African *Manilkara*	21 (27-15)	Africa 97	Oligocene
W	0.99	*Manilkara s.s*. subclade 2	27 (30-23)	Africa 97	Oligocene
X	0.99	Large African *Manilkara*	15 (18-11)	Africa 99	Miocene
Y	0.99	Asian *Manilkara s.s*.	23 (27-19)	Sahul Shelf 52	Oligocene-Miocene

### Ancestral area reconstruction and intercontinental dispersal events

Ancestral area inferences and likelihood support are given in Table 1 and Figure 1, which also indicates the age and direction of inferred dispersal events. The tribe Mimusopeae, subtribe Manilkarinae and the genera *Manilkara, Labramia*, and *Faucherea/Labourdonnaisia* are all inferred to have African ancestry (Figure 1).

Following its origin in Africa during the Oligocene 32 Mya (HPD 36–29; node K) and subsequent diversification 29 Mya (HPD 32–28 Mya; node Q), *Manilkara s.s*. spread via long distance dispersal to Madagascar twice, Asia once and the Neotropics once during the Oligocene–Miocene. Both the *Faucherea/Labourdonnaisia/Manilkara* clade (N) (28 Mya; HPD 33–23 Mya) and the genus *Mimusops* (clade J) (22 Mya; HPD 28–17 Mya) also exhibit a similar pattern, having originated in Africa and later dispersed to both Madagascar and Asia during the Miocene.

Long-distance dispersal from Africa to Madagascar and the surrounding islands has occurred on multiple occasions in the tribe Mimusopeae: twice in *Manilkara s.s*. (X3 and X4, 8–4 Mya); at least once for the clade comprising *Labramia, Faucherea*, and *Labourdonnaisia* between 32 Mya (HPD 36–29; node K) and 30 Mya (HPD 35–26 Mya; node L); and twice in *Mimusops* between 22 Mya (HPD 28–17 Mya; node J) and 9 Mya (HPD 13–5 Mya; node J1), as well as 5 Mya (HPD 2–6 Mya; node J3).

The Neotropical *Manilkara* clade (S) is also derived from an African ancestor, which dispersed to South America during the Oligocene–Miocene between 26 Mya (HPD 30–22 Mya; node R) and 18 Mya (HPD 22–14 Mya; node S). From South America, further dispersal occurred to Central America 16–15 Mya and throughout the Caribbean islands starting from 15 to 10 Mya.

Asia was reached by three independent dispersal events within the tribe Mimusopeae. *Manilkara s.s*. reached Asia from Africa between 27 Mya (HPD 30–23 Mya; node W) and 23 Mya (HPD 27–19 Mya; node Y), while *Mimusops* did the same 8–6 Mya (node J2). The *Manilkara fasciculata* clade reached Asia from Madagascar between 28 (HPD 33–23 Mya; node N) and 15 Mya (HPD 20–10 Mya; node P).

### Diversification rates

**Figure 2:**
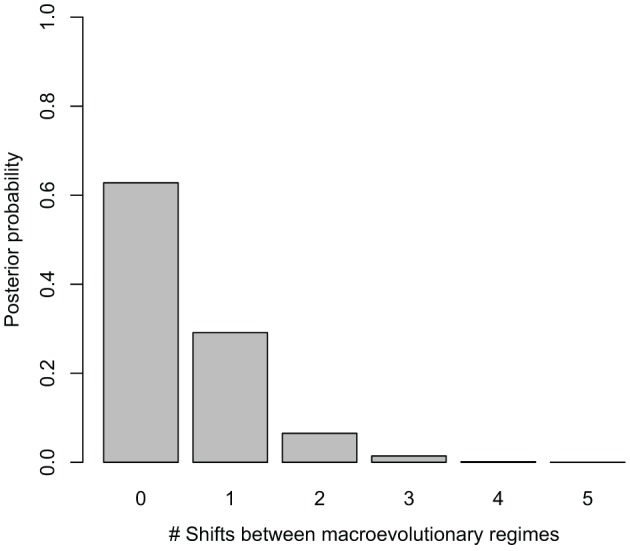
**Posterior probability of models with different number of shifts between macroevolutionary regimes considered in BAMM**. The best models for *Manilkara s.s*. indicate no significant shifts in diversification.

Net diversification rates (lnSR) differed somewhat between regions, ranging from a lowest mean value of 0.06 (0.05–0.07) for the Asian lineage, through 0.10 (0.09–0.10) for the African lineage to a maximum of 0.15 (0.12–0.19) for the Neotropical lineage. Despite sampling models with up to five different macroevolutionary regimes, BAMM analysis selected models without shifts between macroevolutionary regimes along the *Manilkara s.s*. phylogeny, with the highest posterior probability obtained for zero shifts models, i.e., a single, constantly varying net diversification rate throughout the history of the genus (Figure 2). Bayes Factor comparison, following the criteria of Kass and Raftery (1995) provided unsubstantial support (1.68) for the zero shifts models over the models including shifts between macroevolutionary regimes.

**Figure 3:**
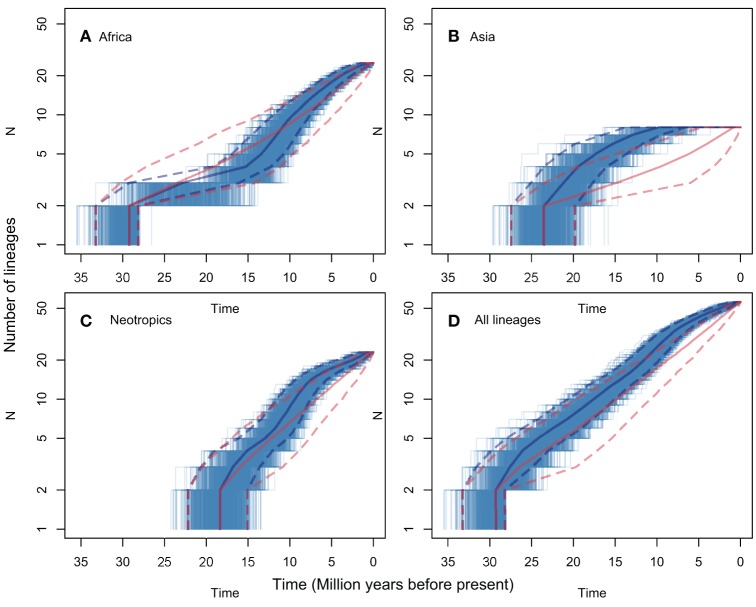
**LTT plots for lineages that included only those species from each of Africa (A), Asia (B), the Neotropics (C), and all species of *Manilkara s.s*. (D)**. Each plot shows the median and 95% HPD of the ages for each number of lineages in solid and dashed lines, respectively. The lines for observed trees are shown in blue and for the trees simulated under a constant diversification process in red. The thinner blue lines correspond to each of the 1000 observed trees. The 95% HPD intervals show major overlap in all plots but non-significant patterns suggest lower diversification rates in part of the histories of African and Asian lineages.

LTT plots are presented in Figure 3, for all regions (Figure 3D) and for the pruned African, Asian and Neotropical lineages (Figures 3A–C respectively). The figure shows both observed rates, and rates predicted for the same numbers of lineages evolving under a constant net diversification rate process (i.e., constant speciation and extinction rates, estimated using BAMM for the genus *s.s*.). None of the observed LTT patterns diverge significantly from those predicted assuming a constant diversification rate. The analyses including all *Manilkara s.s*. lineages (Figure 3D) and only the Neotropical lineage (Figure 3C) both show a good fit between observed patterns and those predicted under a constant diversification rate. In contrast, African lineages (Figure 3A) show a trend toward reduced diversification rates from 25 to 12 Mya, followed by an increase in diversification rates to levels matching those in the Neotropics from 12 Mya to the present. The Asian lineage shows low and decreasing diversification rates toward the present. While the Asian pattern is derived from just eight species, and thus any observed pattern must be interpreted with caution, it is striking that Asia produced no new lineages during the last 7 Mya, at a time when Africa and the Neotropics were both showing rapid diversification.

## Discussion

### Origin of *Manilkara*

The tribe Mimusopeae evolved ~52 Mya (HPD 58–48 Mya; node C) and began to diversify 43 Mya (HPD 44–32 Mya; node D) during the Eocene when global climates were warmer and wetter and a megathermal flora occupied the northern hemisphere. This age estimate also coincides with the first occurrence of putative Mimusopeae fossils recorded from North America and Europe, e.g., *Tetracolporpollenites brevis* (Taylor, 1989), and *Manilkara* pollen (Frederiksen, 1980) in addition to the *Tetracolporpollenites sp*., pollen grain (Harley, 1991), used in this study, which give further weight to the hypothesis that the tribe Mimusopeae was present in the boreotropics and may have originated there. Previous studies (Smedmark and Anderberg, 2007) implicate the break-up of the boreotropics in creating intercontinental disjunctions in the tribe Sideroxyleae and data from the present study are consistent with this hypothesis. Smedmark and Anderberg's (2007) estimate for the age of Sideroxyleae was 68 Mya and in this study the crown node age is reconstructed as being 62 Mya (HPD 73–52 Mya; node B).

The subtribe Manilkarinae evolved 39 Mya (HPD 43–35 Mya; node G), consistent with the hypothesis that it arose late during the existence of the boreotropics. Diversification began 32 Mya (HPD 36–29 Mya; node K), around the time that global cooling and the widening Atlantic were breaking up the boreotropics. Hence migration toward the equator as the climate in the northern hemisphere cooled might have caused or promoted diversification. This transition from the northern hemisphere to equatorial latitudes is also reflected in the putative Manilkarinae fossil record, where during the Oligocene, there is still a strong representation of northern fossils [e.g., Isle of Wight, UK (Machin, 1971), Vermont, USA (Traverse and Barghoorn, 1953; Traverse, 1955) and Czechoslovakia (Prakash et al., 1974)], but fossils also begin to appear in Africa (e.g., *Sapoteae* sp. leaves in Ethiopia, Jacobs et al., 2005). Further cooling and aridification during the Oligocene coincides with diversification of Manilkarinae into genera and may have been a causal factor in this diversification. Alternatively, Manilkarinae may have originated in Africa, as suggested by the ancestral area analysis. However, the analysis cannot account for southward climate shifts and the modern absence of the group from higher latitudes.

*Manilkara* is nested within a grade of other representatives of the tribe Mimusopeae, which is predominantly composed of African taxa (*Mimusops, Tieghemella, Autranella, Baillonella, Vitellaria*, and *Vitellariopsis*) and this suggests that the genus may have had its origin there. In the ancestral area reconstruction both *Manilkara* and the subtribe Manilkarinae are resolved as having a 96% likelihood of an African origin, and the tribe Mimusopeae is reconstructed as having a 99% likelihood of originating in Africa. As such, there is very strong support for an African ancestry for the genus *Manilkara*, the subtribe Manilkarinae and the tribe Mimusopeae.

### The origin of *Manilkara*'s pantropical distribution

Intercontinental disjunctions in *Manilkara* are too young (27–4 Mya) to have been caused by Gondwanan break-up, which would have had to occur before 70 Mya. *Manilkara* is also too young for its pantropical distribution to be the result of migration through the boreotropics, which would have had to occur between 65 and 45 Mya, after which the climate would have been too cool for tropical taxa to cross the North Atlantic Land Bridge, even though this might have persisted until ~33 MYA (Milne and Abbott, 2002). The most likely period for migration of tropical taxa by this route was during the PETM/EECO, 55–50 Mya (Zachos et al., 2001). Furthermore, a boreotropical origin should leave a phylogeographic signature in the form of southern lineages being nested within more northern ones. However, South American lineages are not nested within Central American lineages, and neither are those southeast of Wallace's line nested within those to the northwest. With these vicariance-based explanations not supported, *Manilkara*'s disjunct pantropical distribution could only have resulted from long-distance dispersal from Africa to Madagascar, Asia and the Neotropics. This has been demonstrated for numerous other groups distributed across the tropics, e.g., *Begonia* (Thomas et al., 2012) and *Renealmia* (Särkinen et al., 2007).

*Manilkara* has fleshy, sweet fruit ranging in size from 1.5 to 10 cm, which are consumed by a wide variety of animals. With seeds that are too bulky for wind dispersion, it is more likely that long distance dispersal could have been achieved through transport in the gut-contents of birds or by transoceanic rafting in large mats of vegetation. Houle's (1998) study demonstrated that during the Miocene, intercontinental rafting could have occurred in less than 2 weeks on the North and South Equatorial currents.

### Regional diversification in *Manilkara*

Within the Neotropics, *Manilkara* first colonized South America, as indicated in the reconstruction of the ancestral distribution of clade S. The South American clade (U) is divided into two subclades, which correspond to contrasting regional ecologies, with one clade (U1) comprised of Amazonian species and the other (U2) of Atlantic coastal forest species. The only inconsistency in this geographic pattern is the second accession of *Manilkara cavalcantei* (b), an Amazonian species that the analysis places in the Atlantic coastal forest clade. However, in the plastid tree (Supplementary Figure 1) this accession is resolved in a strongly supported (0.99 pp) Amazonian clade with *M. bidentata, M. huberi*, and *M. paraensis*. The phylogenetic split between these two regions occurred during the Mid-Miocene (12–10 Mya), when the Andes were being elevated (Gregory-Wodzicki, 2000; Graham, 2009) and drainage systems in the Amazon basin began to shift eastwards.

Atlantic coastal species in clade U2 and Amazonian species in clade U1 are geographically separated by the dry biomes of the Cerrado and the Caatinga, as well as the higher relief of the Brazilian shield. Simon et al. (2009) and Fritsch et al. (2004) found that the origin of dry-adapted Cerrado Leguminosae and Melastomataceae lineages span the Late Miocene to the Pliocene (from 9.8 to 0.4 Mya), broadly coinciding with the expansion of C4 grass-dominated savanna biomes. However, it is likely that a dry environment would have been present just prior to this time to allow for adaptation of these groups to the new biome. Such timing is exhibited by the Microlicieae (Melastomataceae), where the crown node is 9.8 Mya, and the stem node is 17 Mya (Fritsch et al., 2004). *Manihot* (Euphorbiacae) species of this biome began to diversify from 6.6 Mya (Chacón et al., 2008). Likewise, a phylogenetic study of *Coursetia* (Leguminosae) (Lavin, 2006) reveals that species which inhabit the dry forest of the Brazilian Caatinga are 5–10 My old. This suggests that the Cerrado and Caatinga could have been in existence, at least in part, by the time the South American *Manilkara* subclades U1 and U2 diverged ca.12 Mya, and their development may have driven the geographical split in this South American lineage of *Manilkara*.

The Central American/Caribbean clade (T) originated following dispersal from South America 16–15 Ma, and then split geographically into a Central American subclade (T1, 6 Ma), and a Caribbean subclade (T2, 11 Ma). The only exception to this geographical structure is the single Central American species, *M. chicle* (T3), which is nested in the Caribbean clade, suggesting a Pliocene dispersal (2 Ma) back to the continent. These age estimates place the New World spread of *Manilkara* prior to the estimated age of the closing of the Isthmus of Panama ~3.5 Ma (Coates and Obando, 1996), although recent studies (Farris et al., 2011) indicate that the Isthmus may have closed much earlier, in which case *Manilkara* may have taken an overland route. Overwater dispersal between Central and South America has been demonstrated in numerous other plant taxa (Cody et al., 2010).

African *Manilkara* species are resolved in two clades, both of which are Oligo-Miocene in age. The main African/Madagascan clade (X) is estimated to be 15 My old (HPD 18–11 Mya), and the smaller clade (V) is 21 My old (HPD 27–15 Mya). Africa has been affected by widespread aridification during the Tertiary (Coetzee, 1993; Morley, 2000). The response by *Manilkara* to this changing climate could have been migration, adaptation or extinction. A study of the rain forest genera *Isolona* and *Monodora* (Annonaceae) found that throughout climatic cycles, taxa remained in remnant pockets of wet forest (Couvreur et al., 2008). They are, therefore, an example of a group that migrated or changed its distribution to track wetter climates. Another study of the genus *Acridocarpus* (Malpighiaceae) (Davis et al., 2002a) indicated an east African dry forest adapted lineage nested within a wet forest lineage. The dry adapted lineage was dated to periods of Oligo-Miocene aridification, and is, therefore, an example of a wet forest lineage, which has adapted to changing environmental conditions rather than becoming restricted to areas of favorable climate. The timing of diversification and evolution of dry-adapted species vs. wet-restricted species in the three African *Manilkara* clades suggests a combination of both scenarios. The split between the African clades occurred between 29 Mya (HPD 32–28 Mya; node Q) and 26 Mya (HPD 30–22 Mya; node R), during a period of dramatic continent-wide cooling, which fragmented the Eocene coast to coast rain forest, potentially isolating the three lineages. A second wave of diversification within the main African/Madagascan clade (X) coincides with the Mid-Miocene climatic optimum 17–15 Mya, when global temperatures warmed (Zachos et al., 2001). During the same period the collision of the African and Eurasian plates closed the Tethys Sea, instigating further aridification. The resulting drier and warmer climates caused the spread of savannas and the retraction of rain forest, as evidenced by an increase in grass pollen during this period (Morley, 2000; Jacobs, 2004). Nonetheless, cladogenesis in the main African/Madagascan clade (X) gained pace from the Mid-Miocene onwards. In particular, a third wave of diversification from rain forest into drier shrubland environments in eastern and southern Africa occurred subsequent to the main uplift of the Tanganyikan plateau in the East African Rift System ca. 10 Mya, which had a significant impact on further regional aridification (Lovett and Wasser, 1993; Sepulchre et al., 2006) (Table 1).

Clade X is predominantly composed of Guineo-Congolian rain forest species. This is almost exclusively the case in subclade X1, aside from the Madagascan taxa, which are also rain forest species. However, within subclade X2, there is a transition from wet to dry environments. The sole Madagascan taxon in this lineage (*M. sahafarensis*) is a dry, deciduous forest species. The four dry, eastern-southern African taxa in subclade X2 (*M. discolor, M. sansibarensis, M. butugi, M. cuneifolia*) all evolved between 8 and 5 Mya subsequent to the main uplift of the East African Rift System. The ancestor of the smaller African clade composed of *M. mochisia* and *M. concolor* also diversified into these two dry-adapted eastern/southern species at the same time 6 Mya (HPD 10–2 Mya). Hence, some African *Manilkara* lineages adapted to a drying climate, while others remained in their ancestral rain forest habitat.

Within the main Asian clade of the plastid phylogeny (Yc1, Supplementary Figure 1), the Indian species *Manilkara roxburghiana* is sister to the other species and the two Fijian species are among the most derived, consistent with the hypothesis that the founding dispersal event was from Africa to India with subsequent spread eastward into Malesia. However, ancestral area reconstruction of the ITS data (node Y, Figure 1) suggests that migration within Asia was from east to west (Sahul Shelf to Sunda Shelf) 23 Mya (HPD 27–19 Mya). Dated phylogenies also indicate that many other angiosperm groups have crossed Wallace's Line from the late Miocene onwards: *Pseuduvaria* (Annonaceae) (Su and Saunders, 2009), Aglaieae (Meliaceae) (Muellner et al., 2008), at least four separate lineages of *Begonia* (Begoniaceae) (Thomas et al., 2012) and *Cyrtandra* (Gesneriaceae) (Cronk et al., 2005). In Sapotaceae four lineages of Isonandreae have migrated from west to east across Wallace's Line (Richardson et al., 2014), whereas evidence from the tribe Chrysophylloideae suggests recent movement in the opposite direction, from Sahul to Sunda Shelf (Swenson et al., 2008). The two youngest (9 Mya) Asian species (*M. vitiensis* and *M. smithiana*) are both Fijian. The oldest land available for colonization in Fiji is between 14 and 5 Mya (Johnson, 1991; Heads, 2006) hence, the age of these two Fijian taxa coincides with the first emergence of land in the archipelago.

### Diversification rates of *Manilkara* in different parts of the tropics

The BAMM analysis did not support significant rate variation among lineages or regions in *Manilkara s.s*. Despite apparent variation in regional patterns revealed by LTT plots (Figure 3), the data most strongly support a model with a single net diversification rate throughout the genus. Trends within the data for specific regions only suggest departure from a constant rate model in Asia and Africa. Given that observed patterns do not exceed the 95% confidence intervals for the constant rate model for either region, these trends must be considered with caution. This is particularly true for Asia, for which the pattern was derived from only eight species. Because sensitivity and statistical power of methods for detection of shifts in diversification rates may correlate positively with the number of species in the clade (Silvestro et al., 2011), rate shifts in clades with a small number of species (as in Asia for *Manilkara s.s*.) may not have been detected by the methods used here (a potential type two error). A simulation study would be required to examine the impact of taxon number on type two error rates in these analyses. Similarly, small numbers of taxa may be more likely to generate apparent trends through stochastic effects, and these could also generate the apparent two-phase pattern of low, and then rapid, diversification in African lineages.

Taken at face value, net diversification rates and LTT plots both suggest a trend for more rapid diversification in Neotropical and African lineages than in Asian ones. The timing of rapid Neotropical diversification falls within the time frame of Andean uplift (i.e., from the late Miocene onwards), proposed as a diversification engine in many taxa (e.g., Richardson et al., 2001). However, because many South American *Manilkara* species are native to the Atlantic Forest, on the opposite side of the continent from the Andes, Andean uplift may be considered unlikely to directly explain high diversification rates region-wide. Interestingly, the rapid diversification of the African lineage coincided with periods of regional aridification. The slowest diversification rate, in the Southeast Asian lineage, includes species that are mostly to the east of Wallace's Line. This may be explained by the fact that the mountainous topography of much of this region (dominated by New Guinea) limits the habitat available for lineages such as *Manilkara* that are largely restricted to lowland rain forest that covers a greater area of Africa or the Neotropics. Although there is no statistical support for significant diversification rate variation in *Manilkara s.s*., the causes highlighted here should have similar impacts on other lowland rainforest taxa—a prediction that can be tested in future studies utilizing phylogenies of more species rich taxa and meta-analyses of multiple unrelated lineages.

## Author contributions

This paper is a result of Kate E. Armstrong's Ph.D. thesis research at the Royal Botanic Garden Edinburgh and University of Edinburgh. Kate E. Armstrong and James E. Richardson conceived the study and Kate E. Armstrong carried out the research and wrote the manuscript apart from the diversification rate analysis, which was conducted and written by Eugenio Valderrama. James E. Richardson, Graham N. Stone, and Richard Milne supervised the Ph.D. project. Graham N. Stone and James E. Richardson edited the manuscript. James A. Nicholls assisted with phylogenetic analyses. Arne A. Anderberg, Jenny Smedmark, Laurent Gautier, and Yamama Naciri contributed DNA sequence data to the study. All authors have reviewed the manuscript.

### Conflict of interest statement

The authors declare that the research was conducted in the absence of any commercial or financial relationships that could be construed as a potential conflict of interest.

## Supplementary material

### Incongruence between nuclear and plastid trees

Phylogenies generated with nuclear (Figure 1) and plastid data (Supplementary Figure 1) showed high topological congruence. However, there are a couple examples of hard incongruence (strongly supported clades which conflict in their placement between the two datasets), both of which have biogeographic implications. The first is in the placement of the two Asian species *Manilkara hexandra* and *M. littoralis*, and the two African species *M. mochisia* and *M. concolor*. In the ITS phylogeny *M. hexandra* and *M. littoralis* are resolved in the Asian clade Y, while *M. mochisia* and *M. concolor* are resolved in the small African clade V. In contrast, in the plastid phylogeny, these four species form a strongly supported clade (posterior probability 1), marked Φ in Supplementary Figure 1.

A second hard incongruence is apparent in the placement of the three taxa *Manilkara yangambensis, M. triflora*, and *M. suarezensis*. In the plastid phylogeny these form a monophyletic clade Z (Supplementary Figure 1). In contrast, in the ITS analysis, the Brazilian *M. triflora* was poorly resolved at the base of clade T, whereas the Madagascan *M. suarezensis* was resolved within the main African clade (X). The Congolese species *M. yangambiensis* was not included in the ITS analysis due to difficulties in amplifying its DNA from herbarium specimens.

These discrepancies between the nuclear and plastid trees may be the result of either ancestral polymorphism with incomplete lineage sorting or chloroplast capture (introgression) following dispersal.

### Evidence for chloroplast capture?

In the dated nuclear phylogeny, the Asian species *M. hexandra* (Sri Lanka) and *M. littoralis* (Myanmar) (clade Y1) are placed with other Asian species (clade Y2), whereas in the plastid phylogeny, they are resolved in clade Φ with two African species *M. mochisia* (Zambia) and *M. concolor* (South Africa) (from clade V in ITS). This suggests hybridization of taxa across the Indian Ocean possibly resulting in chloroplast capture. Intercontinental chloroplast capture may also be implicated in the case of clade Z, which is resolved in the plastid analyses but not in the ITS analyses and is composed of *M. suarezensis* (Madagascar), *M. triflora* (Brazil), and *M. yangambiensis* (Congo). The ITS analysis did not include *M. yangambiensis*, but placed *M. triflora* with other Neotropical species in clade S, and *M. suarezensis* with other Madagascan species within a larger clade of African species (clade X). Therefore, ITS resolved at least two of the clade Z species with species from the same landmass, but cpDNA did not, and resolved them together instead. Clade Z is strongly supported (pp 0.99) in the plastid analysis. Assuming that the correct species level relationships are resolved, clade Z presents a case of long distance dispersal and chloroplast capture more remarkable than the clade V/Y1 scenario, because it involves species from three landmasses, and hence two dispersal events.

Hybridization and chloroplast capture across long distances such as ocean barriers has been indicated previously in Sapotaceae. The species *Chrysophyllum cuneifolium* is inferred to have originated from an intercontinental hybridization event where the chloroplast is South American and the nuclear genome is African (Särkinen et al., 2007). Likewise, the Pacific genus *Nesoluma* is hypothesized to have arisen as a result of intercontinental hybridization in the boreotropical region during the Eocene (Smedmark and Anderberg, 2007). *Nesoluma* presents the opposite pattern to *Chrysophyllum*, where the chloroplast is African and the nuclear genome is Neotropical. Hybridization between New and Old World lineages has also been demonstrated in the pantropical genus *Gossypium* (Malvaceae; Wendel et al., 1995) and intercontinental chloroplast capture is hypothesized to have also occurred in *Thuja* (Cupressaceae; Peng and Wang, 2008). Additionally, both hybridization and introgression events are inferred to have occurred between distantly related species in *Ilex* (Aquifoliaceae; Manen et al., 2010). What is abundantly clear is that long distance dispersal has played a crucial role in the establishment of the modern distribution of *Manilkara*.

Supplementary Figure 1 **Bayesian majority rule consensus tree of the chloroplast dataset**. Posterior probability values are indicated above branches. Nodes with letters/symbols are discussed in the text.

Supplementary Figure 2 **Phylogenetic tree used in the BAMM analysis showing the nodes for which a proportion of sampled species was calculated, as shown in Supplementary Table 3**.

Supplementary Table 1 **Herbarium specimen data, GenBank accession number and ancestral area coding for taxa included in the analyses**. Accessions of newly generated sequences are emboldened.

Supplementary Table 2 **Chloroplast primers designed for this study**.

Supplementary Table 3 **Lineage specific correction used to take into account incomplete taxon sampling in the BAMM analysis**. The unsampled species were assigned to the more recent node including the species with the most similar morphology. The proportion of sampled over total taxa was calculated for the nodes shown in Supplementary Figure 2.
