# Corrigendum: Patterns of diversification amongst tropical regions compared: a case study in Sapotaceae

**DOI:** 10.3389/fgene.2015.00086

**Published:** 2015-03-05

**Authors:** Kate E. Armstrong, Graham N. Stone, James A. Nicholls, Eugenio Valderrama, Arne A. Anderberg, Jenny Smedmark, Laurent Gautier, Yamama Naciri, Richard Milne, James E. Richardson

**Affiliations:** ^1^The New York Botanical GardenNew York, NY, USA; ^2^Institute of Evolutionary Biology, University of EdinburghEdinburgh, Scotland; ^3^Royal Botanic Garden EdinburghEdinburgh, Scotland; ^4^Naturhistoriska RiksmuseetStockholm, Sweden; ^5^University Museum of BergenBergen, Norway; ^6^Conservatoire et Jardin BotaniquesGenève, Switzerland; ^7^Institute of Molecular Plant Sciences, University of EdinburghEdinburgh, Scotland; ^8^Laboratorio de Botánica y Sistemática, Universidad de los AndesBogotá, Colombia

**Keywords:** Sapotaceae, *Manilkara*, pantropical, biogeography, diversification rates

In the sixth paragraph of the Discussion section entitled “Regional Diversification in *Manilkara*,” in the sentence beginning “In Sapotaceae four lineages of Isonandreae have migrated… ” the citation of Swenson et al., 2008 should instead be: Swenson, U., Nylinder, S., and Munzinger, J. (2014). Sapotaceae biogeography supports New Caledonia being an old Darwinian island. *J. Biogeogr.* 41, 797–809. doi: 10.1111/jbi.12246

Additionally, in the second paragraph of the Supplementary Material section entitled “Evidence for Chloroplast Capture?” in the sentence beginning “The species *Chrysophyllum cuneifolium* is inferred to have originated… ” the citation of Särkinen et al., 2007, should instead be: Swenson, U., Richardson, J. E., and Bartish, I. V. (2008). Multi-gene phylogeny of the pantropical subfamily Chrysophylloideae (Sapotaceae): evidence of generic polyphyly and extensive morphological homoplasy. *Cladistics* 24, 1006–1031. doi: 10.1111/j.1096-0031.2008.00235.x

## Conflict of interest statement

The authors declare that the research was conducted in the absence of any commercial or financial relationships that could be construed as a potential conflict of interest.

