# Supplementary material for: Patterns of diversification amongst tropical regions compared: a case study in Sapotaceae
Source: Front Genet. 2014 Dec 3;5:362. doi: 10.3389/fgene.2014.00362 (PMC4253964; doi:10.3389/fgene.2014.00362)
Supplement: Supplementary Figure 1 — Bayesian majority rule consensus tree of the chloroplast dataset. Posterior probability values are indicated above branches. Nodes with letters/symbols are discussed in the text. [file Table1.DOC]

| **Species** | **ITS** | **rpl32-trnL** | **rps16-trnK** | **trnS-trnfM** | **Collector’s name & number** | **Country of origin** | **Area code** |
| --- | --- | --- | --- | --- | --- | --- | --- |
| *Argania spinosa* | AM408056 | - | - | - | Nordenstam 9325 | Morocco | Africa |
| *Aulandra longifolia* | HF542797 | - | - | - | Christensen 1720 | Sarawak, Malaysia | Sunda shelf |
| *Autranella congolensis* | HF542798 | **KM487321** | **KM487411** | **KM487501** | Bokdam 4401 | Congo | Africa |
| *Baillonella toxisperma* | **KM370893** | - | - | - | Bourobou s.n. | Gabon | Africa |
| *Burckella macropoda* | HF542799 | - | - | - | Chase 1359 | Java, Indonesia | Sunda shelf |
| *Capurodendron androyense* | AM408107 | - | - | - | Humbert 28855 | Madagascar | Madagascar |
| *Diploknema butyracea* | HF542818 | - | - | - | Polunin, Sykes & Williams 3975 | Nepal | South Asia |
| *Diploknema oligomera* | HF542819 | - | - | - | Chase 1360 | Java, Indonesia | Sahul shelf |
| *Eberhardtia aurata* | AM408108 | **KM487322** | **KM487412** | **KM487502** | G. Hao 534 Cultivated | South China Bot. Garden | East Asia |
| *Eberhardtia tonkinensis* | AF456258 | - | - | - | Yang,S.-X. unpublished | Yunnan, China | East Asia |
| *Englerophytum natalense* | AY552150 | - | - | - | C. K. 3483 | Tanzania | Africa |
| *Faucherea manongarivensis* | **KM370894** | **KM487323** | **KM487413** | **KM487503** | L. Gautier et al 3910 | Madagascar | Madagascar |
| *Faucherea parvifolia* | **KM370895** | **KM487324** | **KM487414** | **KM487504** | L. Gautier 4163 | Madagascar | Madagascar |
| *Faucherea* sp. | **KM370896** | **KM487325** | **KM487415** | **KM487505** | A. Anderberg 233 | Madagascar | Madagascar |
| *Faucherea thouvenotii* | **KM370897** | **KM487326** | **KM487416** | **KM487506** | L. Gautier 3938 | Madagascar | Madagascar |
| *Inhambanella henriquesii* | HF542823 | **KM487327** | **KM487417** | **KM487507** | de Winter & Vahrmeijer 8536 | South Africa | Africa |
| *Isonandra compta* | HF542824 | - | - | - | Emanuelsson 3039 | Sri Lanka | South Asia |
| *Isonandra perakensis* | HF542825 | - | - | - | Pennington & Wong 10227 | Malaysia | Sunda shelf |
| *Isonandra* sp. | HF542826 | - | - | - | Philcox, Weerasooriya & Weerasekera 10744 | Sri Lanka | South Asia |
| *Labourdonnaisia callophylloides* | **KM370898** | **KM487328** | **KM487418** | **KM487508** | R. Capuron 28171SF | Reunion | Madagascar |
| *Labourdonnaisia madagascariensis* | **KM370899** | **KM487329** | **KM487419** | **KM487509** | R. Capuron 27747SF | Madagascar | Madagascar |
| *Labourdonnaisia revoluta* | **KM370900** | **KM487330** | **KM487420** | **KM487510** | Lorence 1602 | Mauritius | Madagascar |
| *Labramia anakaranaensis* | **KM370901** | **KM487331** | **KM487421** | **KM487511** | L. Gautier 4037 | Madagascar | Madagascar |
| *Labramia costata* | **KM370902** | **KM487332** | **KM487422** | **KM487512** | G. Schatz & A. Gentry 2094 | Madagascar | Madagascar |
| *Labramia louvelii* | **KM370903** | **KM487333** | **KM487423** | **KM487513** | A. Anderberg 245 | Madagascar | Madagascar |
| *Labramia mayottensis* | HF542830 | **KM487334** | **KM487424** | **KM487514** | Labat et al 3309 | Mayotte, Comoros | Madagascar |
| *Letestua durissima* | **KM370904** | - | - | - | L. White 213 | Gabon | Africa |
| *Madhuca crassipes* | HF542831 | - | - | - | Jugah ak. Kudi 23757 | Sarawak, Malaysia | Sunda Shelf |
| *Madhuca hainanensis* | HF542832 | - | - | - | G. Hao 530, Cultivated | South China Bot. Garden | East Asia |
| *Madhuca leucodermis* | **KM370905** | - | - | - | Takeuchi et al. 17858 | New Guinea | Sahul shelf |
| *Madhuca longifolia* | HF542833 | - | - | - | G. Hao 531, Cultivated | South China Bot. Garden | South Asia |
| *Madhuca microphylla* | HF542834 | - | - | - | Fagerlind 4790 | Sri Lanka | South Asia |
| *Madhuca motleyana* | HF542835 | - | - | - | Pennington & Kochummen 10259 | Malaysia | Sunda shelf |
| *Madhuca palembanica* | HF542836 | - | - | - | Triono, Saman & Victobery 11 | Indonesia | Sunda shelf |
| *Madhuca utilis* | HF542837 | - | - | - | Pennington & Asri 10209 | Malaysia | Sunda shelf |
| *Manilkara bella* | **KM370906** | **KM487335** | **KM487425** | **KM487515** | Folli 501 | Brazil | South America |
| *Manilkara bequaertii* | **KM370907** | **KM487336** | **KM487426** | **KM487516** | F. Breteler 15348 | Gabon | Africa |
| *Manilkara bidentata* (a) | **KM370909** | - | - | - | J. Chave M17116014 (Bridge project) | French Guiana | South America |
| *Manilkara bidentata* (b) | **KM370910** | - | - | - | J. Chave P01860330 (Bridge project) | French Guiana | South America |
| *Manilkara bidentata* (c) | **KM370908** | **KM487337** | **KM487427** | **KM487517** | T. Pennington 1203 | Peru | South America |
| *Manilkara boivinii* | **KM370911** | **KM487338** | **KM487428** | **KM487518** | L. Gautier 3278 | Madagascar | Madagascar |
| *Manilkara butugi* (a) | - | **KM487339** | **KM487429** | **KM487519** | D.R. Chaffey 1252 | Ethiopia | - |
| *Manilkara butugi* (b) | **KM370912** | **KM487340** | **KM487430** | **KM487520** | Friis & Vollesen 740 | Sudan | Africa |
| *Manilkara capuronii* | **KM370913** | **KM487341** | **KM487431** | **KM487521** | R. Capuron 11.377SF | Madagascar | Madagascar |
| *Manilkara cavalcantei* (a) | **KM370914** | - | - | - | Smith 605 | Brazil | South America |
| *Manilkara cavalcantei* (b) | **KM370915** | **KM487342** | **KM487432** | **KM487522** | Vicentini et al 527 | Brazil | South America |
| *Manilkara celebica* | - | **KM487343** | **KM487433** | - | Neth. Ind. For. Service bb 30152 | Sulawesi, Indonesia | - |
| *Manilkara chicle* | HF542839 | **KM487344** | **KM487434** | **KM487523** | Castillo et al 2083 | Guatemala | North America |
| *Manilkara concolor* | HF542840 | **KM487345** | **KM487435** | **KM487524** | Swenson & Karis 635 | South Africa | Africa |
| *Manilkara cuneifolia* | **KM370916** | **KM487346** | **KM487436** | **KM487525** | G. McPherson 16792 | Gabon | Africa |
| *Manilkara dawei* | **KM370917** | **KM487347** | **KM487437** | **KM487526** | D.J. Harris 7707 | Central African Republic | Africa |
| *Manilkara decrescens* | **KM370918** | **KM487348** | **KM487438** | **KM487527** | J.D. & E.G. Chapman 6689 | Brazil | South America |
| *Manilkara discolor* | **KM370919** | **KM487349** | **KM487439** | **KM487528** | K. Vollesen 2460 | Tanzania | Africa |
| *Manilkara dissecta* | **KM370920** | **KM487350** | **KM487440** | **KM487529** | M. Gardner TNCA 4012 | New Caledonia | Sahul shelf |
| *Manilkara elata* | **KM370921** | **KM487351** | **KM487441** | **KM487530** | Jardin et al 2277 | Brazil | South America |
| *Manilkara fasciculata* | **KM370922** | **KM487352** | **KM487442** | **KM487531** | K. Armstrong 353 | West Papua, Indonesia | Sahul shelf |
| *Manilkara fouilloyana* | **KM370923** | **KM487353** | **KM487443** | **KM487532** | G. McPherson 16173 | Gabon | Africa |
| *Manilkara gonavensis* | **KM370924** | **KM487354** | **KM487444** | **KM487533** | Ekman 8741 | Haiti | North America |
| *Manilkara hexandra* | **KM370925** | **KM487355** | **KM487445** | **KM487534** | P.L. Comanor 868 | Sri Lanka | South Asia |
| *Manilkara hoshinoi* | **KM370926** | **KM487356** | **KM487446** | **KM487535** | M. Hoshino 2138 | Pulau | Sahul shelf |
| *Manilkara huberi* (a) | **KM370928** | - | - | - | J. Chave NH200061 (Bridge project) | French Guiana | South America |
| *Manilkara huberi* (b) | **KM370927** | **KM487357** | **KM487447** | **KM487536** | O. Poncy 1828 | French Guiana | South America |
| *Manilkara inundata* | **KM370929** | **KM487358** | **KM487448** | **KM487537** | Sothers & Saraiva 22 | Brazil | South America |
| *Manilkara jamiqui* | **KM370930** | **KM487359** | **KM487449** | **KM487538** | Urquiola & Dressler 529 | Cuba | North America |
| *Manilkara kanosiensis* | - | **KM487360** | **KM487450** | - | Neth. Ind. For. Service bb 24311 | Papua New Guinea | - |
| *Manilkara kauki* | **KM370931** | **KM487361** | **KM487451** | **KM487539** | K. Armstrong 379 | Bali, Indonesia | Sunda shelf |
| *Manilkara koechlinii* | **KM370932** | **KM487362** | **KM487452** | **KM487540** | J. Casier 443 | Dem. Republic of Congo | Africa |
| *Manilkara lacera* (a) | **KM370933** | **KM487364** | **KM487454** | **KM487542** | D.J. Harris 8200A | Gabon | Africa |
| *Manilkara lacera* (b) | - | **KM487363** | **KM487453** | **KM487541** | X.M. van der Burgt 40 | Gabon | - |
| *Manilkara letestui* | - | - | - | **KM487543** | J.J. Bos 5604 | Cameroon | - |
| *Manilkara letouzeyi* | **KM370934** | **KM487365** | **KM487455** | **KM487544** | R. Letouzey 4444 | Cameroon | Africa |
| *Manilkara littoralis* | **KM370935** | **KM487366** | **KM487456** | **KM487545** | Maung Gale 14654 | Myanmar | East Asia |
| *Manilkara longifolia* | **KM370936** | **KM487367** | **KM487457** | **KM487546** | Thomas et al 8076 | Brazil | South America |
| *Manilkara lososiana* | **KM370937** | **KM487368** | **KM487458** | **KM487547** | D. Kenfack 625 | Cameroon | Africa |
| *Manilkara mabokeensis* | **KM370938** | **KM487369** | **KM487459** | **KM487548** | D.J. Harris 7164 | Central African Republic | Africa |
| *Manilkara maxima* | **KM370939** | **KM487370** | **KM487460** | **KM487549** | Sant'Ana et al 670 | Brazil | South America |
| *Manilkara mayarensis* | **HF542844** | **KM487371** | **KM487461** | **KM487550** | Ekman 9971 | Cuba | North America |
| *Manilkara mochisia* (a) | **KM370940** | **KM487372** | **KM487462** | **KM487551** | L. Gautier 4171 | Zambia | Africa |
| *Manilkara mochisia* (b) | - | **KM487373** | **KM487463** | **KM487552** | Bidgood et al 2286 | Tanzania | - |
| *Manilkara multinervis* | **KM370941** | **KM487374** | **KM487464** | **KM487553** | Schmidt et al 3274 | Ghana | Africa |
| *Manilkara napali* | - | - | **KM487465** | **KM487554** | F. Schram BW 1636 | West Papua, Indonesia | - |
| *Manilkara obovata* (a) | - | **KM487375** | **KM487466** | **KM487555** | D.J. Harris 7759 | Central African Republic | - |
| *Manilkara obovata* (b) | **KM370942** | **KM487376** | **KM487467** | **KM487556** | GAF Malanda 7 | Republic of Congo | Africa |
| *Manilkara paraensis* | **KM370943** | **KM487377** | **KM487468** | **KM487557** | Zarucchi et al 2526 | Brazil | South America |
| *Manilkara pellegriniana* | **KM370944** | **KM487378** | **KM487469** | **KM487558** | D.J. Harris & M. Fay 1843 | Cameroon | Africa |
| *Manilkara perrieri* | **KM370945** | **KM487379** | **KM487470** | **KM487559** | R. Capuron 28132-SF | Madagascar | Madagascar |
| *Manilkara pleena* | **KM370946** | **KM487380** | **KM487471** | **KM487560** | A. Lioger & P. Lioger 33453 | Puerto Rico | North America |
| *Manilkara pubicarpa* | - | **KM487381** | **KM487472** | **KM487561** | Forest Dept. British Guyana 5860 | Guyana | - |
| *Manilkara roxburghiana* | - | **KM487382** | - | **KM487562** | Matthew & Rajendran 44790 | India | - |
| *Manilkara rufula* | **KM370947** | **KM487383** | **KM487473** | **KM487563** | G. Ignacio & A. Caurenio 37 | Brazil | South America |
| *Manilkara sahafarensis* | **KM370948** | **KM487384** | **KM487474** | **KM487564** | R. Capuron 20.965-SF | Madagascar | Madagascar |
| *Manilkara salzmannii* | **KM370949** | **KM487385** | **KM487475** | **KM487565** | Hatschbach 68541 | Brazil | South America |
| *Manilkara samoensis* | - | **KM487386** | - | **KM487566** | S.J. Whilmee 226 | Samoa | - |
| *Manilkara sansibarensis* | **KM370950** | **KM487387** | **KM487476** | **KM487567** | Abeid 272 | Tanzania | Africa |
| *Manilkara sideroxylon* | **KM370951** | **KM487388** | **KM487477** | **KM487568** | Ekman 16173 | Cuba | North America |
| *Manilkara smithiana* | **KM370952** | **KM487389** | **KM487478** | **KM487569** | A.C. Smith 1450 | Fiji | Sahul shelf |
| *Manilkara* sp. 1 | **KM370953** | - | - | - | F.H. Damon 217 | Papua New Guinea | Sahul shelf |
| *Manilkara* sp. 2 | **KM370954** | - | - | - | P199701118/SO118, Cultivated, Purwodadi B.G. | Indonesia | Sahul shelf |
| *Manilkara staminodella* | **KM370955** | **KM487390** | **KM487479** | **KM487570** | Anderberg et al 50 | Costa Rica | North America |
| *Manilkara suarezensis* | **KM370956** | - | - | **KM487571** | Randriamampionona 248 | Madagascar | Madagascar |
| *Manilkara subsericea* | **KM370957** | **KM487391** | **KM487480** | **KM487572** | Hatschbach & Souza 51302 | Brazil | South America |
| *Manilkara sulcata* | - | - | - | **KM487573** | Frontier-Tanzania Coastal Forest Res. Prog. 1045 | Tanzania | - |
| *Manilkara triflora* | **KM370958** | **KM487392** | **KM487481** | **KM487574** | Fonseca et al 2887 | Brazil | South America |
| *Manilkara udoido* | **KM370959** | **KM487393** | **KM487482** | **KM487575** | S. Slappy LR26622 | Palau | Sahul shelf |
| *Manilkara valenzuelana* | **KM370960** | **KM487394** | **KM487483** | **KM487576** | A. Lioger & P. Lioger 22980 | Dominican Republic | North America |
| *Manilkara vitiensis* | **KM370961** | **KM487395** | **KM487484** | **KM487577** | Smith 1461 | Fiji | Sahul shelf |
| *Manilkara welwitschii* | **KM370962** | **KM487396** | **KM487485** | **KM487578** | van den Houten et al 25 | Gabon | Africa |
| *Manilkara yangambiensis* | - | **KM487397** | **KM487486** | **KM487579** | C. Evrard 1499 | Dem. Republic of Congo | - |
| *Manilkara zapota* | **KM370963** | **KM487398** | **KM487487** | **KM487580** | J. Clayton 12 | Trinidad | North America |
| *Manilkara zenkeri* | **KM370964** | - | **KM487488** | - | Doumenge 526 | Cameroon | Africa |
| *Mimusops caffra* | HF542847 | **KM487399** | **KM487489** | **KM487581** | Swenson & Karis 636 | South Africa | Africa |
| *Mimusops comorensis* | HF542848 | **KM487400** | **KM487490** | **KM487582** | Pignal & Ginguette 1065 | Comoros Islands | Madagascar |
| *Mimusops coriacea* | **KM370965** | - | - | - | Bernadi 11891 | Madagascar | Madagascar |
| *Mimusops elengi* | HF542849 | **KM487401** | **KM487491** | **KM487583** | Chantaranothai 2305 | Thailand | East Asia |
| *Mimusops kummel* | **KM370966** | - | - | - | Kayambo 4996 | Tanzania | Africa |
| *Mimusops lecomtei* | **KM370967** | - | - | - | L. Chatrou 631 | Madagascar | Madagascar |
| *Mimusops membranacea* | **KM370968** | - | - | - | Randrianaivo 126 | Madagascar | Madagascar |
| *Mimusops obovata* | HF542850 | **KM487402** | **KM487492** | **KM487584** | Swenson & Karis 633 | South Africa | Africa |
| *Mimusops perrieri* | **KM370969** | - | - | - | S.F. 18297 | Madagascar | Madagascar |
| *Mimusops* sp. (voalala complex) | **KM370970** | - | - | - | Randrianaivo 583 | Madagascar | Madagascar |
| *Mimusops zeyheri* | HF542851 | **KM487403** | **KM487493** | **KM487585** | Dahlstrand 6386 | South Africa | Africa |
| *Northia seychellana* | HF542853 | **KM487404** | **KM487494** | **KM487586** | L. Chong-Seng s. n. | Seychelles | Seychelles |
| *Palaquium amboinense* | HF542854 | - | - | - | Iuijesundara s.n. | Sri Lanka | South Asia |
| *Palaquium formosanum* | AM408110 | - | - | - | Chung & Anderberg 1421 | Taiwan | East Asia |
| *Palaquium microphyllum* | HF542855 | - | - | - | Pennington, Kochummen & Wong 10222 | Malaysia | Sunda shelf |
| *Palaquium ridleyi* | **KM370971** | - | - | - | P. Wilkie 858 | Borneo, Malaysia | Sunda shelf |
| *Palaquium stenophyllum* | **KM370972** | - | - | - | F. Slik 9592 | Borneo, Indonesia | Sunda shelf |
| *Payena acuminata* | HF542856 | - | - | - | Chase 1368 | Indonesia | Sunda shelf |
| *Payena lucida* | HF542857 | - | - | - | Ambri et al AA1604 | Borneo | Sunda shelf |
| *Sarcosperma laurinum* | AM408055 | **KM487405** | **KM487495** | **KM487587** | Saunders 2000 | Hong Kong | East Asia |
| *Sideroxylon americanum* | AM408060 | - | - | - | Gillis 11576 | Bahamas | North America |
| *Sideroxylon angustum* | AM408061 | - | - | - | Ekman 4034 | Cuba | North America |
| *Sideroxylon beguei* | AM408062 | - | - | - | McPherson et al 14831 | Madagascar | Madagascar |
| *Sideroxylon betsimisarakum* | AM408063 | - | - | - | Schonenberger et al A-102 | Madagascar | Madagascar |
| *Sideroxylon borbonicum* | AM408064 | - | - | - | Bosser 21325 | Reunion | Madagascar |
| *Sideroxylon capiri* | AM408065 | - | - | - | Garcýa 1848 | Mexico | North America |
| *Sideroxylon capuronii* | AM408066 | - | - | - | Capuron 20151-SF | Madagascar | Madagascar |
| *Sideroxylon celastrinum* | AM408067 | - | - | - | Correll 50467 | Bahamas | North America |
| *Sideroxylon confertum* | AM408068 | - | - | - | Ekman 17405 | Cuba | North America |
| *Sideroxylon contrerasii* | AM408069 | - | - | - | Lundell 20793 | Guatemala | North America |
| *Sideroxylon cubense* | AM408070 | - | - | - | Beurton & Mory 927 | Dominican Republic | North America |
| *Sideroxylon floribundum* | AM408071 | - | - | - | Lundell 20263 | Guatemala | North America |
| *Sideroxylon foetidissimum* | AM408072 | - | - | - | Lundin 638 | Cuba | North America |
| *Sideroxylon galeatum* | AM408073 | - | - | - | Friedman 3288 | Rodrigues | Madgascar |
| *Sideroxylon gerrardianum* | AM408074 | - | - | - | Capuron 28826-SF | Madagascar | Madagascar |
| *Sideroxylon grandiflorum* | AM408075 | - | - | - | Friedman et al 2653, | Mauritius | Madagascar |
| *Sideroxylon horridum* | AM408076 | - | - | - | Gutierrez & Nilsson 5 | Cuba | North America |
| *Sideroxylon ibarrae* | AM408077 | - | - | - | Lundell 19752 | Guatemala | North America |
| *Sideroxylon inerme* | AM408078 | - | - | - | Nielsen s.n., Cultivated | Denmark | Africa |
| *Sideroxylon lanuginosum* | AM408079 | - | - | - | Correll & Ogden 28456 | Texas | North America |
| *Sideroxylon lanuginosum* | AF174617 | - | - | - | R.B. Jackson et al 1999 | Texas, U.S.A. | North America |
| *Sideroxylon leucophyllum* | AM408080 | - | - | - | Carter 5706 | Mexico | North America |
| *Sideroxylon lycioides* | AM408081 | - | - | - | Radford et al 11453 | South Carolina, USA | North America |
| *Sideroxylon majus* | AM408082 | - | - | - | Capuron 28185SF | Reunion | Madagascar |
| *Sideroxylon marginatum* | AM408083 | - | - | - | Leyens CV-96–672 | Cape Verde | Africa |
| *Sideroxylon marmulano* | AM408084 | - | - | - | Swenson & Fernandez 581 | Canary Islands | Africa |
| *Sideroxylon mascatense* | AM408085 | - | - | - | Thulin, Beier & Hussein 9774 | Yemen | Middle East |
| *Sideroxylon obovatum* | AM408086 | - | - | - | Garcýa et al 5586 | Dominican Republic | North America |
| *Sideroxylon obtusifolium* | AM408087 | - | - | - | Alvarez et al 28772 | Mexico | North America |
| *Sideroxylon occidentale* | AM408088 | - | - | - | Carter & Sharsmith 4268 | Mexico | North America |
| *Sideroxylon oxyacanthum* | AM408089 | - | - | - | Wood Y/75/388 | Yemen | Middle East |
| *Sideroxylon palmeri* | AM408090 | - | - | - | Palmer 1513 | Mexico | North America |
| *Sideroxylon persimile* | AM408091 | - | - | - | Veliz 99.7038 | Guatemala | North America |
| *Sideroxylon picardae* | AM408092 | - | - | - | Ekman 15576 | Hispaniola | North America |
| *Sideroxylon portoricense* | AM408093 | - | - | - | Mathew 1 | Jamaica | North America |
| *Sideroxylon puberulum* | AM408094 | **-** | - | - | Coode 4121 | Mauritius | Madagascar |
| *Sideroxylon reclinatum* | AM408095 | - | - | - | Traverse 592 | USA | North America |
| *Sideroxylon repens* | AM408096 | - | **-** | - | Greuter & Rankin 24954 | Dominican Republic | North America |
| *Sideroxylon rotundifolium* | AM408097 | - | - | - | Webster et al 8458 | Jamaica | North America |
| *Sideroxylon salicifolium* | AM408098 | - | - | - | Gutierrez & Nilsson 14 | Cuba | North America |
| *Sideroxylon saxorum* | AM408099 | - | - | - | Jongkind 3500 | Madagascar | Madagascar |
| *Sideroxylon sessiliflorum* | AM408100 | - | - | - | Lorence & Edgerley 2706 | Mauritius | Madagascar |
| *Sideroxylon stenospermum* | AM408101 | - | - | - | Stevens 22935 | Nicaragua | North America |
| *Sideroxylon stevensonii* | AM408102 | - | - | - | Lundell & Contreras 19057 | Guatemala | North America |
| *Sideroxylon tambolokoko* | AM408103 | - | - | - | Capuron 22388-SF | Madagascar | Madagascar |
| *Sideroxylon tenax* | AM408104 | - | - | - | Radford & Leonard 11519 | South Carolina | North America |
| *Sideroxylon tepicense* | AM408105 | **-** | **-** | **-** | Gentry 2931 | Mexico | North America |
| *Sideroxylon wightianum* | AM408106 | - | - | - | G. Hao 532, Cultivated | South China Bot. Garden | East Asia |
| *Tieghemella heckelii* | HF542858 | - | - | - | Jongkind 3936 | Ghana | Africa |
| *Vitellaria paradoxa* | HF542867 | **KM487406** | **KM487496** | **KM487588** | Neumann 1512 | Benin | Africa |
| *Vitellariopsis cuneata* | HF542868 | **KM487407** | **KM487497** | **KM487589** | Thomas 3662 | Tanzania | Africa |
| *Vitellariopsis dispar* | **KM370973** | **KM487408** | **KM487498** | **KM487590** | Pentz 2 | South Africa | Africa |
| *Vitellariopsis kirkii* | HF542869 | **KM487409** | **KM487499** | **KM487591** | Robertson 4085 | Kenya | Africa |
| *Vitellariopsis marginata* | HF542870 | **KM487410** | **KM487500** | **KM487592** | Chase 1122 | South Africa | Africa |
| *Xantolis cambodiana* | AY552155 | - | - | - | Chantaranothai 2507 | Thailand | East Asia |
| *Xantolis siamensis* | AY552154 | - | - | - | Smitairi 1 | Thailand | East Asia |
