# Supplementary material for: Patterns of diversification amongst tropical regions compared: a case study in Sapotaceae
Source: Front Genet. 2014 Dec 3;5:362. doi: 10.3389/fgene.2014.00362 (PMC4253964; doi:10.3389/fgene.2014.00362)
Supplement: Supplementary Figure 2 — Phylogenetic tree used in the BAMM analysis showing the nodes for which a proportion of sampled species was calculated, as shown in Supplementary Table 3. [file Table2.DOC]

Supplementary Table 2. Chloroplast primers designed for this study.

| **Primer name** | **Direction** | **Primer sequence (5’-3’)** |
| --- | --- | --- |
| rpl32-trnL-intF | forward | TCGTCGAGATTGAAGAGTCA |
| rpl32-trnL-intR | reverse | TCTCTTTTGACCGGAAATTCA |
| rpl32_trnL_int_2_F | forward | GGCGGCTGCTCAACTTAT |
| rpl32_trnL_int_2_R | reverse | TCTCTTTTGACCGGAAATTCA |
| rps16-trnK-intF | forward | TGTTCCTGCTATTCTATATTTCCTTG |
| rps16-trnK-intR | reverse | GATGTGTAGATACAATCAGAATCAAAA |
| rps16_trnK_int_2_F | forward | GGGTGCTCAACCTACAGAAA |
| rps16_trnK_int_2_R | reverse | ACGAGGCAATCAAAACATTG |
| trnS-trnFM_int.F | forward | ACTCAGCCATCTCTCCGAAA |
| trnS-trnFM_int.R | reverse | TTTGGGGTGAGAGGAAAAGA |
| trnS-trnFM_int_2_F | forward | AACCACTCAGCCATCTCTCC |
| trnS-trnFM_int_2_R | reverse | GAACCCCTACACTATCACGG |
