# Supplementary material for: Patterns of diversification amongst tropical regions compared: a case study in Sapotaceae
Source: Front Genet. 2014 Dec 3;5:362. doi: 10.3389/fgene.2014.00362 (PMC4253964; doi:10.3389/fgene.2014.00362)
Supplement: Supplementary Table 1 — Herbarium specimen data, GenBank accession number and ancestral area coding for taxa included in the analyses. Accessions of newly generated sequences are emboldened. [file Table3.DOCX]

| **Sampled species** | **Node** | **Proportion sampled** | **Unsampled species** |
| --- | --- | --- | --- |
| *Letestua durissima* | a | 1 |  |
| *Manilkara bella* | d | 0.66 |  |
| *Manilkara bequaertii* | a | 1 |  |
| *Manilkara bidentata* | c | 0.57 | *Manilkara pubicarpa* |
| *Manilkara bovinii* | l | 0.8 |  |
| *Manilkara butugi* | j | 0.66 |  |
| *Manilkara capuronii* | l | 0.8 |  |
| *Manilkara cavalcantei* | a | 1 |  |
| *Manilkara chicle* | f | 0.66 |  |
| *Manilkara concolor* | b | 0.66 | *Manilkara nicholsonii* |
| *Manilkara cuneifolia* | a | 1 |  |
| *Manilkara dawei* | k | 0.6 | *Manilkara frondosa* |
| *Manilkara decrescens* | a | 1 |  |
| *Manilkara discolor* | a | 1 |  |
| *Manilkara elata* | a | 1 |  |
| *Manilkara fouillayana* | a | 1 |  |
| *Manilkara gonavensis* | a | 1 |  |
| *Manilkara hexandra* | h | 0.66 | *Manilkara roxburghiana* |
| *Manilkara hoshinoi* | i | 0.57 |  |
| *Manilkara huberi* | c | 0.57 | *Manilkara bolivarensis* |
| *Manilkara inundata* | c | 0.57 | *Manilkara excelsa* |
| *Manilkara jamiqui* | g | 0.66 | *Manilkara excisa* |
| *Manilkara kauki* | i | 0.57 | *Manilkara celebica, Manilkara kanosiensis* |
| *Manilkara koechlinii* | k | 0.6 | *Manilkara yangambiensis* |
| *Manilkara lacera* | a | 1 |  |
| *Manilkara letouzei* | a | 1 |  |
| *Manilkara littoralis* | h | 0.66 |  |
| *Manilkara longifolia* | a | 1 |  |
| *Manilkara mabokeensis* | a | 1 |  |
| *Manilkara maxima* | e | 0.66 |  |
| *Manilkara mayarensis* | a | 1 |  |
| *Manilkara mochisia* | b | 0.66 |  |
| *Manilkara multinervis* | a | 1 |  |
| *Manilkara obovata* | a | 1 |  |
| *Manilkara paraensis* | c | 0.57 |  |
| *Manilkara pelligriniana* | a | 1 |  |
| *Manilkara perrieri* | l | 0.8 |  |
| *Manilkara pleeana* | g | 0.66 |  |
| *Manilkara rufula* | e | 0.66 | *Manilkara dardanoi* |
| *Manilkara sahafarensis* | j | 0.66 | *Manilkara sulcata* |
| *Manilkara salzmanni* | d | 0.66 | *Manilkara multifida* |
| *Manilkara sansibarensis* | a | 1 |  |
| *Manilkara sideroxylon* | f | 0.66 | *Manilkara spectabilis* |
| *Manilkara smithiana* | i | 0.57 |  |
| *Manilkara staminodella* | a | 1 |  |
| *Manilkara suarezensis* | l | 0.8 | *Manilkara letestui* |
| *Manilkara subsericea* | a | 1 |  |
| *Manilkara triflora* | a | 1 |  |
| *Manilkara valenzuelana* | a | 1 |  |
| *Manilkara vitiensis* | i | 0.57 | *Manilkara samoensis* |
| *Manilkara welwitschii* | k | 0.6 |  |
| *Manilkara zapota* | a | 1 |  |
| *Manilkara zenkeri* | a | 1 |  |
