# Supplementary figures and images for: Patterns of diversification amongst tropical regions compared: a case study in Sapotaceae
Source: Front Genet. 2014 Dec 3;5:362. doi: 10.3389/fgene.2014.00362 (PMC4253964; doi:10.3389/fgene.2014.00362)

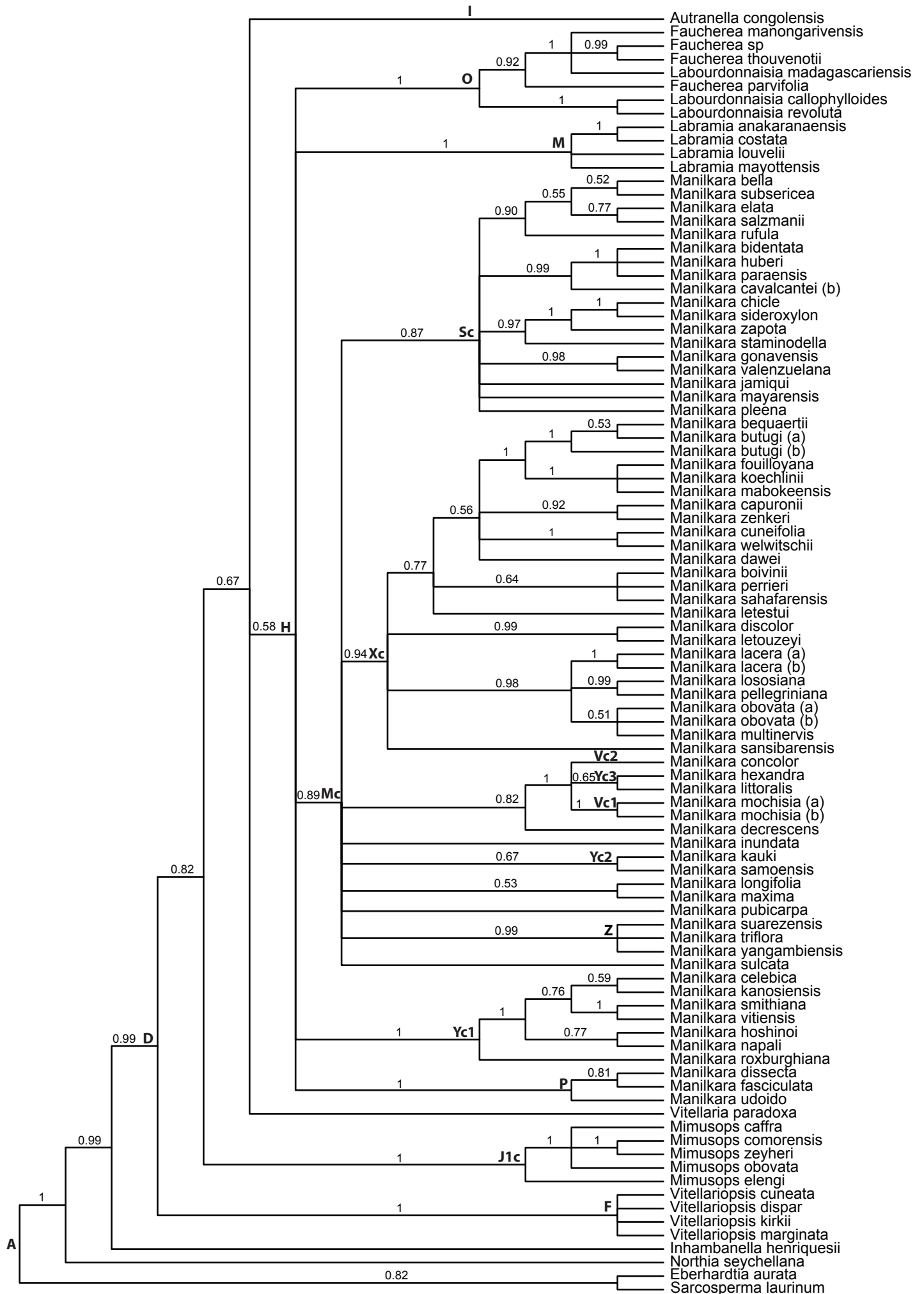

Supplement: Supplementary Table 2 — Chloroplast primers designed for this study. [file Image1.PDF]

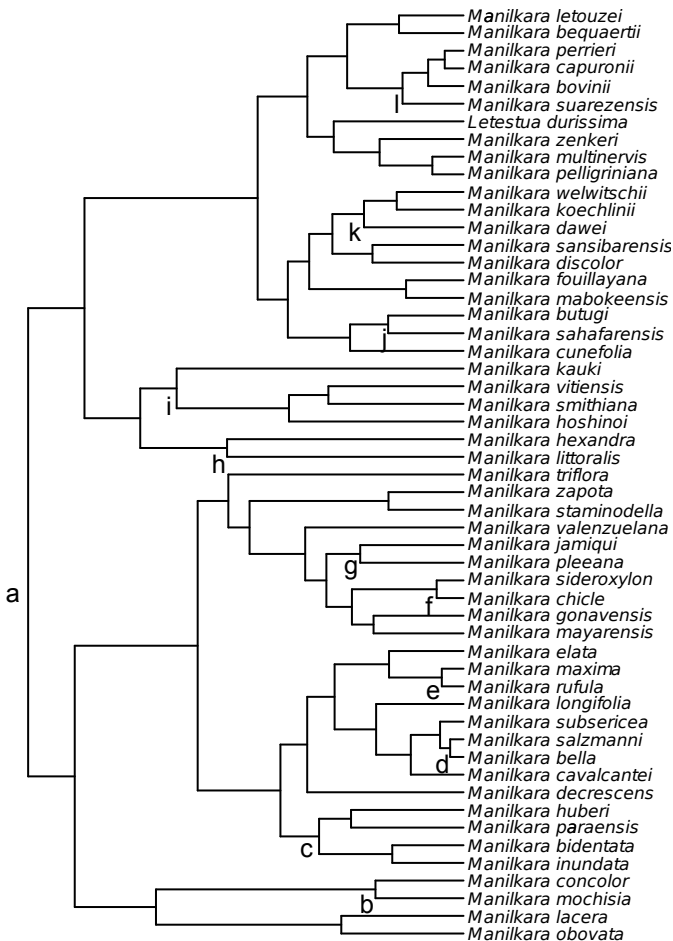

Supplement: Supplementary Table 3 — Lineage specific correction used to take into account incomplete taxon sampling in the BAMM analysis. The unsampled species were assigned to the more recent node including the species with the most similar morphology. The proportion of sampled over total taxa was calculated for the nodes shown in Supplementary Figure 2. [file Image2.PDF]
